# Angiopoietin-1 Promotes the Integrity of Neovascularization in the Subcutaneous Matrigel of Type 1 Diabetic Rats

**DOI:** 10.1155/2019/2016972

**Published:** 2019-01-09

**Authors:** ShaoBin LI, HeQin Zou, MinMin Gong, YuQin Chen, XiaoYong Yan, LiMei Yu, YiBin Yang

**Affiliations:** ^1^Key Laboratory of Cell Engineering of Guizhou Province, The Affiliated Hospital of Zunyi Medical College, Zunyi, Guizhou, China; ^2^Department of Nephrology & Rheumatology, The Affiliated Hospital of Zunyi Medical College, Zunyi, Guizhou, China

## Abstract

**Objective:**

This study aimed to investigate the effects of Ang-1 on neovascularization of diabetic organs by subcutaneous Matrigel angiogenesis model, established in type 1 diabetic rats.

**Methods:**

Ang-1 adenoviral vector was constructed. The rat model was established by STZ and divided into four group. The Matrigel was inserted subcutaneously into the abdominal cavity of rats at 8 weeks, the treatment group was injected with Ang-1 adenovirus vector via tail vein, and the rats were sacrificed at 10 weeks. Neovascularization of Matrigel was observed with transmission electron microscopy. The marker of vascular endothelial cell and pericyte were detected by immunofluorescence. Immunohistochemical detection of the neovascular endothelial junction protein was performed. RT-PCR was used to determine protein expression of neovascular in Matrigel.

**Results:**

Vascular cavity-like structure could be seen in subcutaneous Matrigel of diabetic rats, and the cavity was filled with a lot of red blood cells. Transmission electron microscopy showed that neovascular endothelial structure of the Matrigel was incomplete, while the Ang-1 treatment group had more vascular cavity-like structures, intact vascular endothelial structure, and reduced inflammatory cell infiltration in Matrigel. Additionally, the integrity of vascularization improved, and the marker of pericyte and the cell tight junctions protein was upregulated in Ang-1 treatment group.

**Conclusion:**

Hyperglycemia could induce pathological angiogenesis in subcutaneous Matrigel of diabetic rats, and Ang-1 could upregulate the expression of intercellular junction protein in subcutaneous Matrigel of diabetic rats and promote the integrity of neovascularization in the subcutaneous Matrigel of diabetic rats.

## 1. Introduction

Diabetes mellitus is a metabolic disease whose incidence is rapidly increasing year by year. According to the International Diabetes Federation, there were 415 million adults diagnosed with diabetes worldwide in 2015. In 2040 adult diabetics will increase to 642 million [1]. Long-term hyperglycemia leading to chronic microvascular disease is one of the major complications of diabetes, including diabetic retinopathy, nephropathy, and neuropathy [2]. There was evidence that neovascularization was increased in diabetic nephropathy, retinopathy, and atherosclerosis. The new blood vessels might be immature and leaking which could be caused by abnormal expression of angiogenic factors [3]. Angiogenesis involves a succession of regulatory factors, requiring activation of a series of receptors and ligands to maintain balance between the stimulatory and inhibitory signals [4].

The formation of new vascular networks requires initiation by a proangiogenic growth factor such as vascular endothelial growth factor (VEGF). VEGF is a well‐studied growth factor that effectively promotes neovessel sprouting and growth in the initial phase of angiogenesis. Its high angiogenic potential made scholars believe that VEGF monotherapy may be sufficient to promote therapeutic angiogenesis [4]. However, although VEGF monotherapy was successful in promoting the formation of blood vessels, it causes increased vascular permeability and increases inflammatory penetration of new vessels, leading to the fragility of newly formed vessels [5]. Studies have found that anti-VEGF therapy could improve diabetic nephropathy and retinopathy [6, 7]. However, in recent years, more and more basic and clinical studies have found that anti-VEGF might lead to proteinuria [8], deterioration of renal disease, and renal thrombotic microvascular disease [9], and there was no clinical improvement in most patients with retinopathy [10]. This indicates that a certain amount of VEGF is required to maintain organ function in physiological state. Hence, some scholars have proposed the use of VEGF and Ang-1 combination therapy.

Angiopoietins, as one of the endothelial growth factors, modulates vascular development and remodeling during angiogenesis and inflammation process. The angiopoietin (Ang)/Tie-2 and vascular endothelial growth factor (VEGF) system are two types of vascular regulatory molecules that are crucial for vessel formation and maturation [11, 12]. The Ang-1 showed its capacity through the activation of the Tie2 receptor to modulate the maturation and stabilization of newly formed vessels; in addition, it included adherence and maintenance of the survival of vascular epithelial cells [13]. Physiological and pathological conditions of angiogenesis could be mediated by the coordinated expression of Ang-1/Tie-2 and VEGF [14]. The function of Ang is complementary with VEGF [15]. It may result in the later stages of vascular disappearance if the Ang-1 is downregulated. It is thought to be associated with the neovascular bleeding tendency, plasma leakage, and endothelial cell apoptosis or necrosis. Exogenous administration of Ang-1 could mediate the interaction between endothelial cells and pericytes to promote angiogenesis and stabilize the microvascular endothelial cells in diabetic rats [16]. The combination of VEGF and Ang-1 may be an ideal treatment for microangiopathy

The process of diabetes affecting vascular injury and repair was different from other vascular diseases. There was a paradox of vascular survival in the involved tissues and organs. Promoting the maturation and stabilization of new blood vessels may be an effective treatment. However, it is difficult to observe the neovascularization in vivo. Matrigel has been used to analyze the angiogenic capacity of growth factors, cytokines, chemokines, and non-protein mediators in a number of the different fields [17]. Hence, Matrigel angiogenic model was used, such that intuitive assessment of neovascularization in diabetic conditions, the mechanism of Ang-1 promoting angiogenesis, was explored by expression in Matrigel.

## 2. Materials and Methods

### 2.1. Animals

Sprague-Dawley (SD) rats (n = 40, male, 3~4 months old, 210±20g) in a specific pathogen-free grade were selected randomly. These animals were purchased from the Animal Center of the Third Military Medical University (production license: SCXK Yu 2012-0005). The animal studies were performed after receiving the approval of the Institutional Animal Care and Use Committee (IACUC) of Zunyi Medical College.

### 2.2. Build Adenovirus Vectors and Matrigel Preparation

Human Ang-1 gene was cloned into the adenovirus shuttle plasmid (pDC315-GFP) to get the recombinant plasmid pDC315-GFP-Ang-1. PDC315-GFP-Ang-1 and adenovirus backbone vector (PbhgloxΔE1,3Cre) were cotransfected into 293 cells by Lipofectamine 2000 (Thermo Fisher Scientific, America) with the aid of a specific recombination system (Cre/loxP), amplified, and purified to produce the recombinant adenoviral vector Ad-Ang-1 with Ang-1 gene titrated to 2.5 × 10^10^ pfu/ml (Genechem, Shanghai, China).

Sterilized Matrigel was soaked in PBS or 100*μ*l of VEGF_165_ (PeproTech, NJ) overnight at 4°C, placing the syringes, EP tubes, and so on into the refrigerator at 4°C precool overnight.

### 2.3. Treatment of Animals

The rat model with diabetes was established by STZ (Sigma, America). Before STZ was dissolved in 0.l mol/L, pH 4.2-4.5 sterile citric acid-sodium citrate buffer, the final concentration was 1% STZ, by a single intraperitoneal injection of 55mg/kg body weight. Peripheral blood from the tail vein was then tested to confirm diabetes induction after 72h. Blood glucose >16.6mmol was measured for three consecutive days as a model standard [18]. The blood glucose (72h, 8W, 10W) and urine protein (2W,10W) were observed. Under anesthesia with 5% chloralic hydras, Matrigel was inserted subcutaneously through 1 cm orthogonal incisions in the abdomen of the animals, and a lump is formed after solidification [19].

The subjects were randomly designated into four groups of 10 rats each. Group (1) was the normal control group; normal SD rats were injected with Matrigel with a tail vein injection of citrate buffer. Group (2) was the diabetes group; diabetic rats were injected with Matrigel with a tail vein injection of citrate buffer. Group (3) was the blank vector group; while injecting Matrigel into diabetic rats, at the same time 1 × 10^8^ pfu blank adenovirus vector was injected into a tail vein, and the same operation was conducted one week later. Group (4) was the Ang-1 treatment group; while injecting Matrigel into diabetic rats, at the same time 1 × 10^8^ pfu Ang-1 adenovirus was injected into a tail vein, and the same operation was conducted one week later.

The rats were sacrificed under anesthesia at day 14 postprocedure. Opening the abdomen of the rat subcutaneously, carefully separating the subcutaneous Matrigel, the Matrigel was properly stored.

### 2.4. Immunocytochemical Staining Analysis

The harvested Matrigel implants were fixed in 10% formalin solution, embedded into paraffin blocks, and sectioned sagittally (5*μ*m). Renal histopathological changes were assessed microscopically with hematoxylin (ZSBIO, Beijing, China) and eosin staining (ZSBIO, Beijing, China).

For immunohistochemical staining, Matrigel paraffin block was cut into 5*μ*m thickness, xylene dewaxing, and alcohol hydration. After hydration, dropping blocking antibody, a panel of primary antibodies against rat ZO-1 (1:100 ZSBIO China), occludin (1:400 Abcam Britain), Connexin40 (1:400 Abcam Britain), and VE-cadherin (1:400 Abcam Britain) were used. The activity of endogenous peroxidase was quenched by incubation with hydrogen peroxide (3%) for 5 min, followed by microwave treatment for 5 min in citrate buffer for antigen retrieval. The sections were incubated with primary antibodies at 4°C overnight. The next day, the sections were incubated with primary anti-rabbit antibody for 30min. The color was developed in DAB, stained with hematoxylin, and observed under a microscope. The picking conditions were determined. Five fields (× 200) were collected randomly from each section. The average integral optical density (IOD) was measured by Image-Pro Plus 6.0 (Media Cybernetics, America) for semiquantitative analysis.

### 2.5. Immunofluorescence Histochemistry

Matrigel was removed from the liquid nitrogen, embedded in OCT solution, cut into 3-4*μ*m sections with a cryostat, fixed with paraformaldehyde for 60min, and finally washed with PBS. Dropping blocking antibody for 20min, a panel of primary antibodies against rat *α*-SMA (1:200 Abcam Britain), JG12 (1:50 Sant Cruz America), and desmin (1:200 Abcam Britain) were used. The sections were incubated with primary antibodies at 4°C overnight. Rinsed with PBS, the corresponding fluorescent secondary antibody was added dropwise and incubated at 37°C for 70min. The negative control was set in each experiment, and PBS buffer instead of primary antibody was used as negative control. Expression and distribution of fluorescence were observed under IX51 inverted fluorescence microscope (Olympus Japan).

### 2.6. Transmission Electron Microscope

Matrigel was removed from the 2.5% glutaraldehyde fixative solution, fixed with 1% osmium tetroxide for 2h, respectively, and dehydrated with ethanol. Acetone mixture of epoxy resin was soaked and then immersed in pure epoxy resin 3h, waiting for aggregation into blocks. The blocks were cut into 3-4*μ*m thickness, and ultrathin sections were stained with uranyl acetate and lead citrate for observation using H-600A-2 transmission electron microscope (Hitachi, Japan).

### 2.7. Semiquantitative RT-PCR

The frozen Matrigel, which had been added to the Trizol solution, was homogenized fully. An equal amount of chloroform was added to the tissue. 0.5 ml of the supernatant was added followed by precipitation with isopropanol after washing with DEPC water and finally dissolving in 0.1% DEPC water. The purity and concentration of RNA in the sample were determined by spectrophotometry, A260/A280 (1.6-2.0). Using a thermocycler and the first strand cDNA Synthesis Kit, the total RNA was reverse-transcribed into cDNA, in compliance with the manufacturer's protocol. Relevant primer sequences were found from GenBank. Primer design was designed and synthesized by Shanghai Bioengineering Co., Ltd. (Table 1). A total of 20 *μ*l of the amplification reaction system was set up, including 2 *μ*l of cDNA template, 0.8 *μ*l of upstream primer, 0.8 *μ*l of downstream primer, 0.4 *μ*l of ROX Reference Dye, 10 *μ*l of SYBR Premix Ex Taq II (2 ×), and 6 *μ*l of ddH2O. The reaction conditions were as follows: predenaturing at 95°C for 30 sec, PCR at 95°C for 5 sec, and annealing at 60.3°C for 20 sec, total of 35 cycles, and finally at 55°C for 10 s. Result analysis and treatment were performed using 2^−ΔΔCt^ relative quantification method [20].

### 2.8. Statistical Analysis

Data are presented as mean ± standard error (SEM). All statistical analyses were performed using SPSS 18.0 software (SPSS, Chicago, IL, USA), and one-way ANOVA was conducted for comparisons between groups, with P<0.05 considered to be statistically significant.

## 3. Results

### 3.1. Ang-1 Promotes Subcutaneous Matrigel Angiogenesis in Diabetic Rats

**Figure 1:**
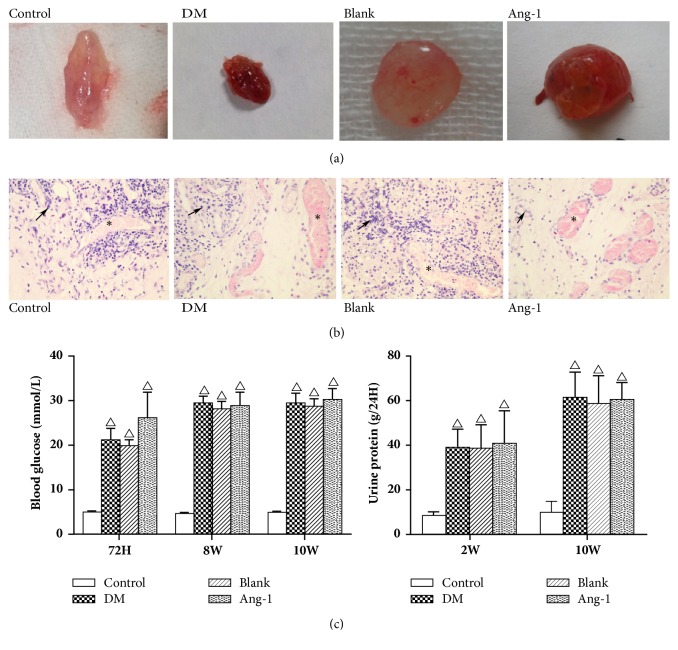
Effect of Ang-1 on neovascularization in diabetic rats. (a) Observe the appearance of the Matrigel. (b) H&E staining of Matrigel. Black arrows represent inflammatory cells, *∗* represents neovascularization, ×200. (c) STZ-induced type 1 diabetes in rats with blood glucose and urinary protein, where △ represents P <0.05 compared with the control group.

Ang-1 cannot significantly change the blood glucose in STZ-induced type 1 diabetic rats (Figure 1(c)). From the blood glucose and urine protein, it can be seen that the experimental diabetes model is established, and there is a trend of diabetic nephropathy [21].

Matrigel was taken out from the rats and compared with respect to the morphology of Matrigel with each other. There was a small amount of angiogenesis observed in the normal group of Matrigel (Figure 1(a)), the Matrigel in the diabetic group was carnation and moist with the visible vascular network. In addition, the treatment group Matrigel was deep red, showing large neovascularization; the morphology is more regular than others.

In order to assess the effect of Ang-1 on angiogenesis, to monitor vascularization, and to examine the angiogenic microenvironment in the Matrigel, the study performed histological analysis using HE staining. There was a few neovessels in normal group Matrigel (Figure 1(b)), and a large number of neovessels could be seen in all three groups in diabetic Matrigel, showing the tube-like structure. Inflammatory cell infiltration was more severe in the control group than in the experimental group. The number of angiogenic neovessels observed in Ang-1 group was more compared to the other groups.

### 3.2. Ang-1 Enhances Microvascular Ultrastructure and Vessel Integrity

**Figure 2:**
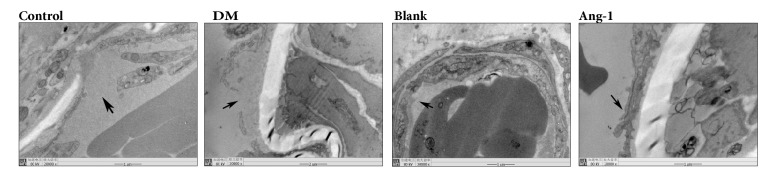
Electron micrographs of the newly formed microvessels in the Matrigel. Ultrastructural characterization of microvessels in the diabetes group of Matrigel; the microvessels have poorly organized basement membrane and swollen endothelial cells, as well as damaged endothelial tissue (Black arrow). Few pericytes are distributed around the endothelium, magnification ×20000. Ang-1 treated Matrigel showed complete endothelial structure with the typical tight junction (black arrows). Pericytes were observed to be in direct contact with endothelial cells. Multiple elongated pericytes are embedded within the intact basement membrane, magnification ×20000.

A transmission electron microscope was used to observe the ultrastructural features of microvascular Matrigel. The normal group vascular structure was incomplete. The structure of microvessels was abnormal in the diabetic group and blank adenovirus group. Endothelial cells are swollen, in which chromatin and mitochondria degenerate, and the endothelial structure is vague with local lesions damaged (Figure 2). Compared with the subcutaneous Matrigel of Ang-1 adenovirus group, the endothelial structure of neovasculature was intact under the electron microscope, and the endothelial cells were arranged in a monolayer to form tight junctions. The star-shaped pericytes continuously and tightly wrap blood vessels, forming a clear basement membrane with endothelial cells. It shows that Ang-1 could recruit endothelial cells and pericytes, favoring the formation of neovessels.

### 3.3. Neovascular Endothelial Cells and Pericytes Related Markes Expression

**Figure 3:**
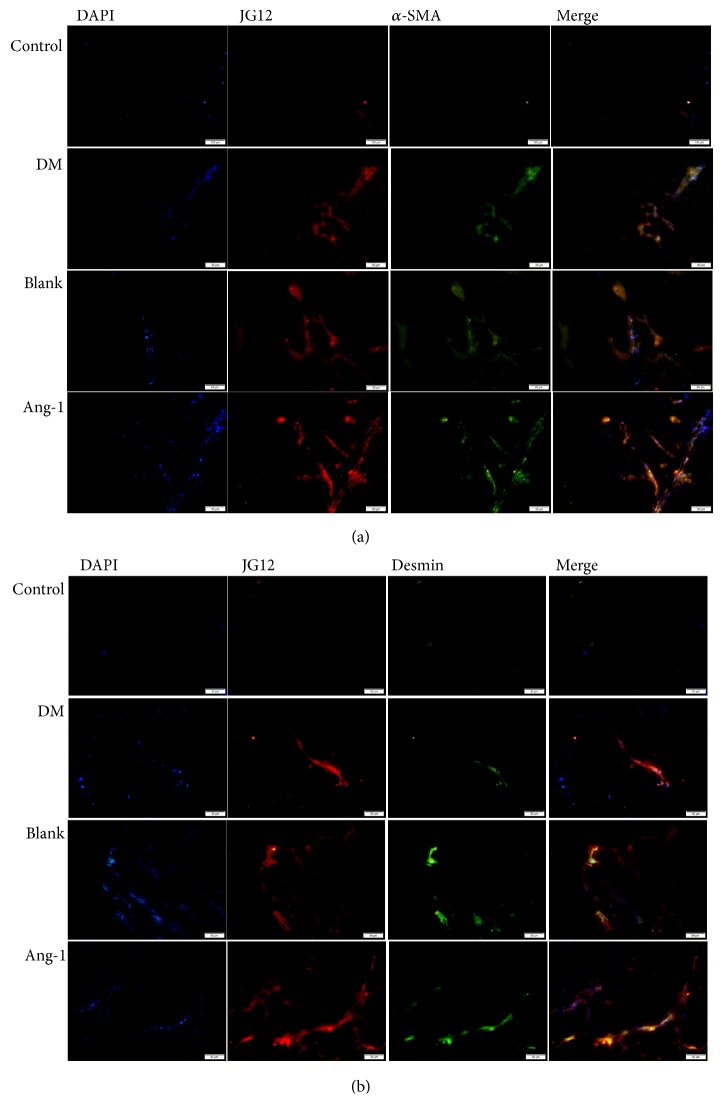
Immunofluorescence analysis of pericyte coverage of microvessels in the Matrigel. (a) Ang-1 treatment of diabetic rats; the neovascular marker JG12 (red) and the expression of desmin (green), a pericyte marker, were confirmed by immunofluorescence, 50*μ*m. (b) New angiogenic marker JG12 (red) and pericyte *α*-SMA (green) expression were confirmed by double-labeling technique, 50*μ*m.

The study was aimed at investigating the effect of Ang-1 on angiogenesis in a diabetic model. In order to monitor the formation of neovascularization in Matrigel, immunofluorescence was mainly used to detect the expression of JG12. An endothelial cell marker protein and the expression of pericyte markers of *α*-SMA and desmin in different groups were used to explain the problem [22]. The JG12 positive expression could be clearly detected (Figure 3(a)). At the same time, green-stained *α*-SMA and desmin-positive pericytes were observed (Figure 3). In the Ang-1 group, the pericytes were densely packed with endothelial cells, whereas only some of the microvessels in the control group were covered by pericytes. Almost every microvessel was colocalized with pericytes in the experimental group, whereas only some of the microvessels were covered with pericytes in the control group.

### 3.4. Expression Changes of ZO-1, Occludin, VE-Cadherin, and Connexin40 after Ang-1 Treatment

**Figure 4:**
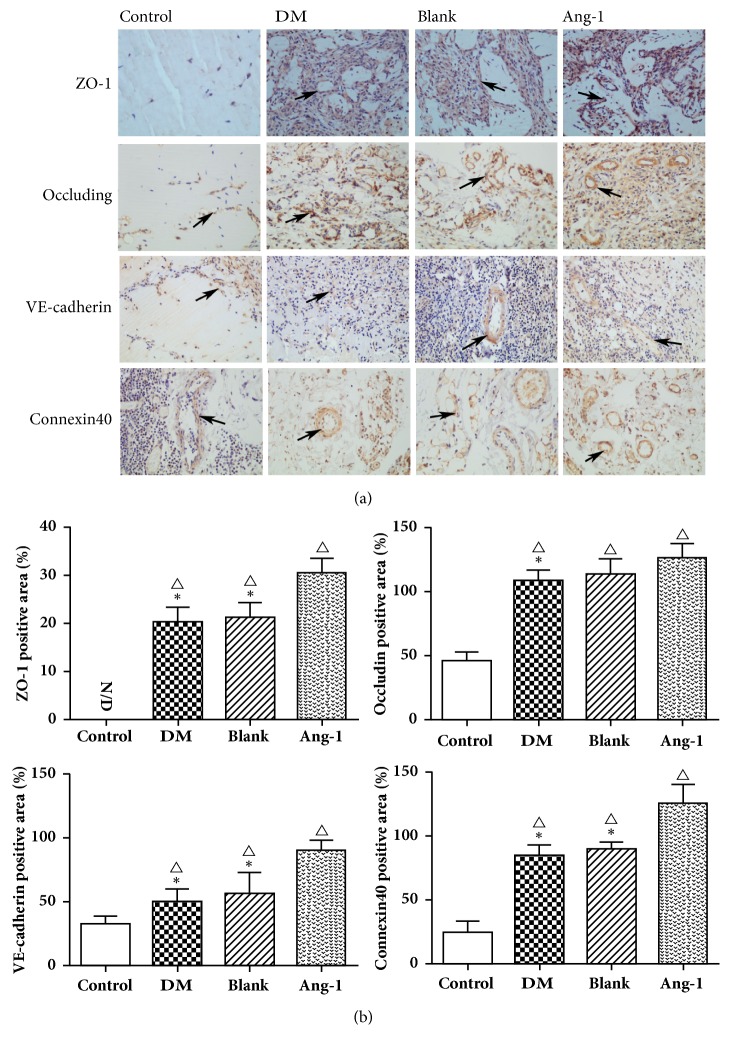
Vascular maturity-related protein expression after Ang-1 treatment. (a) Immunohistochemical staining of ZO-1, occludin, VE-cadherin, and Connexin40 in the four groups was performed. Several proteins are expressed on the envelope of endothelial cells (black arrow), × 200. (b) The relative quantification of ZO-1, occludin, VE-cadherin, and Connexin40 in four groups. △ represents P <0.05 compared with the control group, *∗* represents P <0.05 compared with the Ang-1 treatment group, and N/D represents no expression.

Vessel maturation and stability is the key to angiogenesis, and vascular stability mainly depends on the tight junctions between endothelial cells. Tight junctions consist of different types of transmembrane proteins and complex cytoplasmic proteins [23]. Therefore, in order to observe the effect of Ang-1 on the neovascularization of diabetic model, the study used immunohistochemistry to observe the expression of ZO-1, occludin, VE-cadherin, and Connexin40 to judge the vascular maturation (Figure 4). ZO-1 is mainly expressed on the cell envelope and plasma, and almost no expression in the normal group was observed, while the other three groups indicated different levels of expression (Figure 4(a)). The expression of ZO-1 in Ang-1 group was significantly higher than that in the diabetic group and blank vector group (P <0.05). The tight junction of the two key transmembrane components, occludin and Connexin40, expression is also similar to ZO-1, and expression of protein in Ang-1 treatment group was upregulated (P<0.05). There was no statistically significant difference between the diabetic group and the blank vehicle group (P>0.05).

VE-cadherin is a major molecule for adhesion junctions of vascular endothelial cells. It is also an endothelial cell-specific cadherin, essential for the maintenance of vascular endothelial cell polarity and vascular integrity, through the end of the cytoplasmic domain and intracellular catenin to form a complex and function [24]. ZO-1 is activated by VE-cadherin regulation of endothelial cell junctions, formed in regulating the actin cytoskeleton and during the formation of functional endothelial cells. The VE-cadherin protein is mainly expressed in the membrane and cytoplasm. The results were also the same as expected; the expression of VE-cadherin in the Ang-1 treatment group was significantly upregulated compared with the other two groups (P<0.05), consistent with the growth trend of ZO-1.

### 3.5. Real-Time PCR Analysis

**Figure 5:**
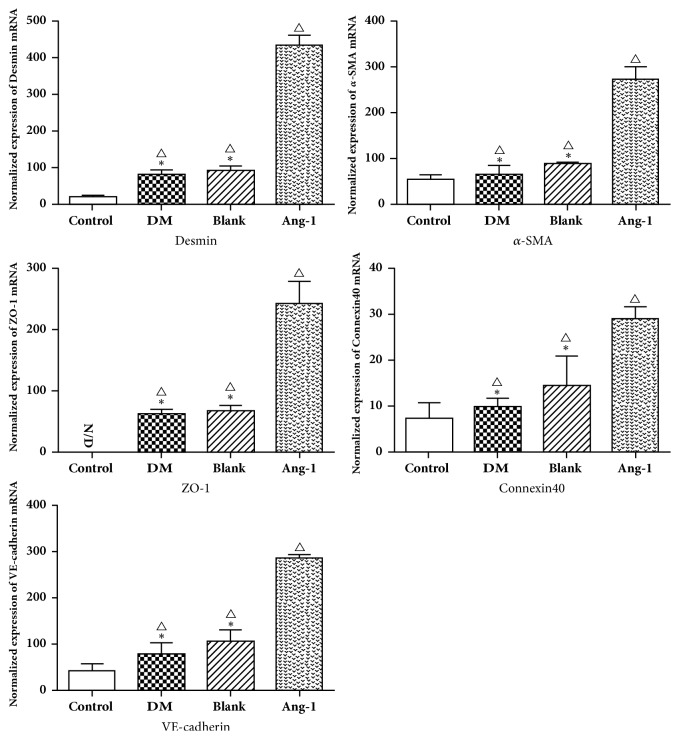
Analysis of Vascular maturity-related protein expression by RT-PCR. The mRNA expression of *α*-SMA, desmin, ZO-1, VE-cadherin, and Connexin40 was detected. △ represents P <0.05 compared with the control group, *∗* represents P <0.05 compared with the Ang-1 treatment group, and N/D represents no expression.

In addition, the changes in gene expression of *α*-SMA, desmin, ZO-1, occludin, and Connexin40 after Ang-1 treatment were quantitatively analyzed by qRT-PCR. The expression of proteins and their genes varies between different groups. The expression of *α*-SMA and desmin mRNA was gradually increasing, significantly higher than that in the diabetic group and the blank adenovirus group including the normal control group, and the difference observed was statistically significant (P<0.05) (Figure 5). The mRNA of Zo-1, occludin, and Connexin40 also showed similar expression, significantly higher than the normal control group, diabetic group, and blank adenovirus group, showing a gradual upward trend; the difference was statistically significant (P<0.05) (Figure 5). The mRNA expression trend was basically the same as that of immunohistochemistry.

## 4. Discussion

Diabetic microangiopathy can severely impact survival times and life quality of the patients, and its pathogenesis is complex; the treatment is limited, so it has received much attention from the industry [25]. Long-term high glucose will accelerate arterial remodeling, atherosclerosis, and endothelial cell (EC) dysfunction, which has affected the macro- and micro-circulatory systems, resulting in tissue progressive hypoperfusion ischemia and hypoxia. Microvascular systems in diabetic patients have impaired antioxidant defenses due to increased production of reactive oxygen species (ROS) and the breakdown of mitochondria, leading to dysfunction of vascular endothelial cells [26]. Many studies have demonstrated the high expression of VEGF in early diabetic organ tissues and its association with pathological neovascularization [27]. VEGF treatment alone can effectively promote the generation and growth of new vessels at the beginning of angiogenesis; however, the lack of vascular basement membrane, *α*-smooth muscle actin (*α*-SMA), and pericyte has led to the degeneration of the new vessels [28]. Ang-1 can promote the interaction between vascular EC and pericyte, thus resulting in vascular maturing [29]. Angiopoietin/Tie and VEGF/VEFDR are the two major signaling pathways that synergistically regulate angiogenesis in both physiological and pathological conditions [30]. It is difficult to assess the neovascularization of organ tissues (especially the kidneys) in vivo. We chose to construct a Matrigel angiogenesis model and transplanted it into subcutaneous of diabetic rat. We simulated the high VEGF expression of diabetic organ tissue by preadding VEGF to Matrigel. The effect of Ang-1 on Matrigel neovascularization was assessed by high expression of Ang-1 adenovirus by tail vein injection.

To investigate the effect of Ang-1 on neovascularization, we first observed the neovascular structure from electron microscopy. Transmission electron microscope results indicate incomplete EC structure and local damage in both DM group and blank adenovirus group. The main reasons for the increased vascular endothelial permeability are the breakdown of endothelial cell junctions and the integrity of the endothelial barrier [31]. High glucose environment can promote aggravate organ and tissue oxidative stress and increase the production of reactive oxygen species (ROS), which leads to endothelial dysfunction [32]. Factors such as only upregulated renal VEGF expression lead to immature angiogenesis which will result in increased vascular permeability [33]. The Ang-1 adenovirus group exhibited intact EC structure and typical intercellular tight junction. It contributes to the improvement of microvascular permeability under DM status and maintains the stability of new vessels. Meanwhile, an obvious tubular structure filled with numerous red blood cells can also be observed from the HE pathological sections of Ang-1 group; the other groups had more inflammatory cells and fewer neovascularity. Interestingly, a large number of inflammatory cells are found in three DM models, which may be partly induced by the exogenous implantation of Matrigel. Ang-1 can suppress EC apoptosis and relieve oxidative stress, both under high glucose environment [34]. Gamble JR et al. found that angiopoietin-1 inhibits TNF-alpha-stimulated leukocyte transmigration. Angiopoietin-1 may have a major role in maintaining the integrity of endothelial monolayers [35]. Combined with the experimental results, it showed the effect of Ang-1 on stabilizing the new vessels.

In order to observe the effect of Ang-1 with VEGF on the production of new vessels on type I diabetic rats, the EC specific marker aminopeptidase P (JG12) is adopted to evaluate the changes in Matrigel angiogenesis. JG12 can bind with aminopeptidase P on EC, but not with lymphatic EC, which is considered to be a vascular EC marker more reliable than CD34 [36]. These are consistent with other studies; that is, diabetic microangiopathy, the common pathway, is a progressive loss of the microvascular system, resulting in kidney tissue or other organ tissue fibrosis. In a sense, capillary rarefaction is a crucial contributor to the pathophysiology of diabetic microangiopathy [37]. The results suggest increased new vessels in Matrigel, indicating successful modeling of the subcutaneous Matrigel angiogenesis model of diabetic rat, which has also established a foundation for subsequent studies. So, the angiogenesis effect of Ang-1 may contributes to favorable parameters. The disturbance in peripheral capillary may be caused by abnormal vessel structure in Matrigel due to glycation end products in hyperglycemia. However, mature microvascular system is not just the presence of endothelial cells. Pericytes and endothelial cells constitute a barrier between microvessels and tissues, which is the main factor for maintaining internal environment stability.

Pericytes adhere to the abluminal surface of endothelial tubules and are required for the formation of stable vascular networks. In capillary, the pericyte can modulate vascular permeability and vascular diameter, deliver the contractility, and maintain vascular stability as well as EC survival through the cell-matrix and cell-cell interactions [38]. High glucose causes migration of peritubular pericytes away from the capillary into the interstitial space. The capillary becomes destabilized, resulting in microvascular rarefaction. Therefore, the changes of pericytes can be observed through neovascularization integrity [39]. Research suggests that the common pericyte markers include *ɑ*-SMA, desmin, and NG2 [40]. In the current study, we adopt the marker combination of *ɑ*-SMA with desmin to observe the distribution of pericyte. The results of immunofluorescence and RT-PCR expression suggest that *α*-SMA and desmin in Ang-1 treatment group were upregulated, and the pericyte wraps the EC to form the tight junction. One of the earliest changes observed in retinal capillaries affected by diabetic retinopathy (DR) is the loss of pericytes; this may make the capillaries vulnerable. According to several reports, exposure of pericytes to high glucose levels reduced their proliferation and induced apoptosis [41]. By upregulating VEGF expression alone, it has been shown to induce pericyte ablation in the mature vasculature [42]. In our experimental results, the pericyte expression in the diabetic group was downregulated, which was contrary to the expression in the Ang-1 group. Exogenous administration of Ang-1 treatment led to increase in the number of pericytes. Ang-1 expression appears to have a critical role in angiogenesis, pericyte recruitment, and vascular stabilization [43]. This is in line with our experiment.

In different organ tissues and pericytes have different contact relationships with vascular endothelial cells, such as tight junctions, adhesive junctions, nail-groove complexes, and focal adhesions [44]. Our study further demonstrated the effects of Ang-1 on the tight junction protein ZO-1, occludin, and adherens junction protein VE-cadherin, Connexin40 expressions. We evaluated the tight junction and adhesion junctions of endothelial and pericytes in hyperglycemic environments, which resulted in downregulating expression of four junction proteins. There are research findings that diabetic hyperglycemia was associated with a decrease in the cell‐cell tight junction and downregulation of VE-cadherin, ZO-1, and occludin protein expression exposure to the abnormal glucose concentration [45, 46]. The reason for this finding could be that disrupted calcium dependence of EC‐EC connections via VE‐cadherin, morphological rearrangement, and paracellular gap formation was induced after exposure to high glucose [47]. Meanwhile, our data also indicate that exogenous administration of Ang-1 can upregulate expression of junction proteins ZO-1, occludin, and VE-cadherin in the Matrigel of diabetic rat. There are studies that have shown that Ang-1 protects blood-brain barrier permeability and cerebral infarct size by upregulating ZO-1, occludin, and VE-cadherin [48]. Siddiqui MR et al. found that Ang-1 inhibits thrombin induced tyrosine phosphorylation of occludin and promotes occludin interaction with ZO-1 to stabilize tight junction [49]. Meanwhile, Ang-1 could block VEGF induced endothelial permeability by inhibiting Src mediated phosphorylation of VE-cadherin [50]. In addition, high glucose treatment caused a decrease in Connexin40 protein expression in ECs and impaired endothelial capillary network formation, which was restored by Connexin40 overexpression [51]. Gene knockout of Connexin40 leads to a reduction in vascular growth and capillary density in the neovascularization model of the mouse neonatal retina [52]. Data indicate that Ang-1 treatment can upregulate the expression of Connexin40 in hyperglycemic environment and thus improve endothelial cell connectivity and reduce vascular permeability. Thus Ang-1 may have a role in upregulating the Connexin protein between endothelial cells and pericytes. It is indicated that Ang-1 plays an important role in maintaining microvascular barrier integrity.

## 5. Conclusion

In summary, we indirectly confirm the existence of angiogenesis in diabetic organ tissues from another perspective by observing the model of diabetic subcutaneous stromal angiogenesis. Ang-1 is a vascular growth factor, the combined application of which with VEGF in subcutaneous Matrigel in the diabetic rat can promote angiogenesis. More importantly, it can give rise to vascular maturation and stabilization. In this experiment, the subcutaneous Matrigel angiogenesis model of diabetes rats was successfully constructed and therapeutic research was carried out to lay a foundation for the follow-up treatment of diabetic microangiopathy.

## Figures and Tables

**Table 1:** Real-time PCR primer sequences.

**Gene symbols**	**Protein name**	**Forward primer(5'-3')**	**Reverse primer(5'-3')**
*GAPDH*	GAPDH	GGAGATTACTGCCCTGGCTCCTA	GACTCATCGTACTCCTGCTTGCTG
*CDH5*	VE-cadherin	CAGCAACTTCACCCTCATCA	GCACAGGCAGGTAGTGGAAC
*gja5*	Connexin40	GGTCCCTCTGCTGGTATTTG	ATAGCGTCGGTGTCAGGAAT
*Acta2*	*ɑ*-SMA	AGGGAGTGATGGTTGGAATG	GGTGATGATGCCGTGTTCTA
*LOC108267036*	Desmin	CCATTGCGGCTAAGAACATC	TTCCATCATCTCCTGCTTGG
*Tjp1*	ZO-1	TGCTCCAGCAGGTCCTAAGT	TGGTAGCTGAGGGCAGAACT

## Data Availability

The data used to support the findings of this study are available from the corresponding author upon request.
